# Clinical implications of high NQO1 expression in breast cancers

**DOI:** 10.1186/1756-9966-33-14

**Published:** 2014-02-05

**Authors:** Yang Yang, Yan Zhang, Qunying Wu, Xuelian Cui, Zhenhua Lin, Shuangping Liu, Liyan Chen

**Affiliations:** 1 Department of Pathology & Cancer Research Center, Yanbian University Medical College, Yanji 133002, China; 2 Department of Breast Surgery, The Second Hospital of Jilin University, Changchun 130041, China; 3 Department of Pathology, Yanbian Women and Children’s Hospital, Yanji 133002, China; 4 Department of Biochemistry, Yanbian University Medical College, Yanji 133002, China

**Keywords:** Breast cancer, NQO1, Immunohistochemistry, Prognosis

## Abstract

**Background:**

NAD (P) H: quinone oxidoreductase 1 (NQO1) is a xenobiotic metabolizing enzyme that detoxifies chemical stressors and antioxidants, providing cytoprotection in normal tissues. However, high-level expression of NQO1 has been correlated with numerous human malignancies, suggesting a role in carcinogenesis and tumor progression. This study aimed to explore the clinicopathological significance of NQO1 and as a prognostic determinant in breast cancer.

**Methods:**

A total of 176 breast cancer patients with strict follow-up, 45 ductal carcinoma *in situ* (DCIS), 22 hyperplasia and 52 adjacent non-tumor breast tissues were selected for immunohistochemical staining of NQO1 protein. Immunofluorescence staining was also performed to detect the subcellular localization of NQO1 protein in MCF-7 breast cancer cells. Eight fresh breast cancers paired with adjacent non-tumor tissues were quantified using real time RT-PCR (qRT-PCR) and western blot. The correlations between NQO1 overexpression and the clinical features of breast cancer were evaluated using chi-square test and Fisher’s exact tests. The survival rate was calculated using the Kaplan–Meier method, and the relationship between prognostic factors and patient survival was also analyzed by the Cox proportional hazards models.

**Results:**

NQO1 protein showed a mainly cytoplasmic staining pattern in breast cancer. The strongly positive rate of NQO1 protein was 61.9% (109/176) in breast cancer, and was significantly higher than in DCIS (31.1%, 14/45), hyperplasia tissues (13.6%, 3/22) and adjacent non-tumor tissues (13.5%, 7/52). High-level expression of NQO1 protein was correlated with late clinical stage, poor differentiation, lymph node metastasis, Her2 expression and disease-free and 10-year overall survival rates in breast cancer. Moreover, multivariate analysis suggested that NQO1 emerged as a significant independent prognostic factor along with clinical stage and Her2 expression status in patients with breast cancer.

**Conclusions:**

High-level expression of NQO1 appears to be associated with breast cancer progression, and may be a potential biomarker for poor prognostic evaluation of breast cancers.

## Background

Breast cancer is one of the most common malignancies in women worldwide and the second leading cause of cancer death among women [1,2]. Studies over the past several decades have found that the expression profiles of estrogen receptor (ER), progesterone receptor (PR) and human epidermal growth factor receptor 2 (Her2)/neu are closely related with breast cancer, and have been used for predicting the outcome and response to breast cancer therapy [3,4]. Although numerous advances in prevention, surgical resection, and adjuvant chemoradiotherapy have led to a decline in the overall mortality due to breast cancer, the survival rates for patients with metastatic disease have not significantly improved [5,6]. Consequently, the discovery of novel biomarkers involved in the diagnosis and progression of breast cancer is of great value.

NAD (P) H: quinone oxidoreductase 1 (NQO1), also known as DT-diaphorase, menadione reductase, or quinone reductase 1, is a cytoplasmic flavoenzyme encoded by a gene located on chromosome 16q22. NQO1 uses NADH or NADPH as substrates to directly reduce quinones to hydroquinones [7,8]. Functions of NQO1 include xenobiotic detoxification, superoxide scavenging and the maintenance of endogenous antioxidant vitamins [9]. It is conceivable that NQO1 plays an important role in protecting normal cells against oxidative injury and carcinogenesis. Paradoxically, despite the cellular functions of this “cell protector”, the antioxidant role of NQO1 was suggested by evidence that the disruption of the NQO1 gene or genetic polymorphism increased the risk of chemical-induced toxicity and cancers [10,11]. NQO1 has been found to be expressed at high levels in many human tumors, including breast cancer, melanoma, lung cancer, cholangiocarcinoma and pancreatic cancer [12-15]. In addition, the high level of NQO1 expression in solid tumors in combination with the ability to reduce many quinine-containing antitumor drugs has dawn attention to NQO1 as a potential molecular target in cancer treatment [16,17]. However, the clinical significance of NQO1 expression status in breast cancer remains unclear.

In this study, we demonstrated the clinicopathological significance of NQO1 through prognostic evaluation of NQO1 overexpression in breast cancers. The results revealed that NQO1 protein is frequently upregulated in breast cancers compared with hyperplasia and adjacent non-tumor breast tissues. These findings indicate that NQO1 may be a good independent predictor of prognosis for patients with breast cancer.

## Materials and methods

### Ethics statement

This research complied with the Helsinki Declaration and was approved by the Human Ethics Committee and the Research Ethics Committee of Yanbian University Medical College. Patients were informed that the resected specimens were stored by the hospital and potentially used for scientific research, and that their privacy would be maintained. Follow-up survival data were collected retrospectively through medical record analyses.

### Clinical samples

Eight fresh breast cancers paired with adjacent non-tumor tissues were snapfrozen in liquid nitrogen and stored at -80°C until use. The histopathology of each specimen was reviewed on the hematoxylin and eosin-stained tissue section to confirm diagnosis and tumor content at least 70% of tumor cells in the tissue sample. The study of 176 paraffin embedded breast cancer samples, as well as 45 ductal carcinoma *in situ* (DCIS) samples, 22 hyperplasis and 52 adjacent non-tumor tissues were also conducted. These samples were selected randomly from patients who underwent surgery between 2002 and 2009, with strict follow-up for survival status. Clinicopathological classification and staging were determined according to the American Joint Committee on Cancer (AJCC) criteria. Clinical information of the samples is summarized in Table 1.

**Table 1:** Correlation between NQO1 protein expression and the clinicopathological parameters of breast cancer

**Variables**	**No. of cases**	**NQO1 strongly positive cases (%)**	***χ***^***2***^	***P*** **value**
**Age**			0.751	0.386
≥50	94	61 (64.9%)		
<50	82	48 (58.5%)		
**Menopausal status**			1.159	0.282
premenopausal	72	48 (66.7%)		
Postmenopausal	104	61 (58.7%)		
**Tumor size**			3.033	0.082
T1	97	51 (52.6%)		
T2	89	58 (65.2%)		
**Histological grade**			11.298	0.004**
Grade-1	82	40 (48.8%)		
Grade-2	51	37 (72.5%)		
Grade-3	43	32 (74.4%)		
**Clinical stage**			7.050	0.008**
0-II	104	56 (53.8%)		
III-IV	72	53 (73.6%)		
**LN metastasis**			7.710	0.005**
Absent	74	37 (50.0%)		
Presence	102	72 (70.6%)		
**ER**			0.614	0.423
Positive	101	60 (59.4%)		
Negative	75	49 (65.3%)		
**PR**			1.426	0.232
Positive	103	60 (58.3%)		
Negative	73	49 (67.1%)		
**Her2 status**			5.534	0.019*
Positive	96	67 (69.8%)		
Negative	80	42 (52.5%)		

*p<0.05 and **p<0.01.

### Immunohistochemical (IHC) analysis

IHC analysis was performed using the DAKO LSAB kit (DAKO A/S, Copenhagen, Denmark). Briefly, to eliminate endogenous peroxidase activity, 4 μm thick tissue sections were deparaffinized, rehydrated and incubated with 3% H_2_O_2_ in methanol for 15 min at room temperature (RT). The antigen was retrieved at 95°C for 20 min by placing the slides in 0.01 M sodium citrate buffer (pH 6.0). The slides were then incubated with the NQO1 monoclonal antibody (1:200, A180: sc-32793, Santa Cruz Biotechnology, Santa Cruz, CA, USA) at 4°C overnight. After incubation with the biotinylated secondary antibody at RT for 30 min, the slides were incubated with a streptavidin-peroxidase complex at RT for 30 min. IHC staining was developed using 3,3′-diaminobenzidine, and Mayer’s hematoxylin was used for counterstaining. We used tonsil sections as the positive control and mouse IgG as an isotope control. In addition, tissue sections were processed omitting the primary antibody as the negative control.

Two pathologists (Lin Z & Liu S) who did not possess knowledge of the clinical data examined and scored all tissue specimens. In case of discrepancies, a final score was established by reassessment by both pathologists on a double-headed microscope. Briefly, the IHC staining for NQO1 was semi-quantitatively scored as ‘–’ (negative) (no or less than 5% positive cells), ‘+’ (5–25% positive cells), ‘++’ (26–50% positive cells) and ‘+++’ (more than 50% positive cells). The cytoplasmic expression pattern was considered as positive staining. Tissue sections scored as ‘++’ and ‘+++’ were considered as strong positives (high level expression) of NQO1 protein.

### Immunofluorescence (IF) staining analysis

IF staining was used to detect the sub–cellular localization of NQO1 protein in MCF-7 breast cancer cells. All steps were performed at RT. MCF-7 cells were grown on coverslips to 70–80% confluence, then fixed with 4% paraformaldehyde for 10 min and permeabilized with 0.5% TritonX-100 for 10 min after 24 h. After blocking with 3% Albumin Bovine V (A8020, Solarbio, Beijing, China) for 1 h, the slides were quickly and gently washed with PBS. The cells were then incubated with the NQO1 antibody (1:500) at 4°C overnight, and followed by incubation with Alexa Fluor® 568 goat anti-mouse IgG (H + L) (A11004, 1:1000, Invitrogen, Carlsbad, CA, USA) for 1 h. After washing with PBS, cells were counterstained with 49-6-diamidino-2-phenylindole (DAPI) (C1006, Beyotime, Shanghai, China) and the coverslips were mounted with Antifade Mounting Medium (P0126, Beyotime) [18]. Finally, the IF signals were visualized under a Leica SP5II CLSM microscope (Heidelberg, Germany) with filters for the corresponding fluorescent stains.

### Western blotting

Fresh tissue samples were ground to powder in liquid nitrogen and lysed with SDS-PAGE sample buffer. Equal protein samples (20 μg) were separated on 10.5% SDS polyacrylamide gels and transferred to PVDF membranes (Immobilon P, Millipore, Bedford, MA, USA). Membranes were blocked with 5% fat-free milk in phosphate-buffered saline with Tween-20 for 1 h at RT. Membranes were incubated with the NQO1 antibody (1:1000) overnight at 4°C, and then with horseradish peroxidase-conjugated goat anti-mouse IgG (CWBIO, China, CW0096A). NQO1 expression was detected using ECL Prime western blotting detection reagent (Amersham) according to the manufacturer’s instructions. Anti-β-actin mouse monoclonal antibody (CW0096A CWBIO, China) was used as a loading control [19].

### Quantitative real-time PCR (qRT-PCR)

As described previously [20], total RNA samples from eight of primary tumor materials were extracted using Trizol reagent (Invitrogen, Carlsbad, CA, USA) according to the manufacturer’s instructions. The extracted RNA was pretreated with RNase-free DNase, and 2 μg RNA from each sample was used for cDNA synthesis primed with random hexamers. For the PCR amplification of NQO1 cDNA, an initial amplification step using NQO1 specific primers was performed with denaturation at 95°C for 15 min, followed by 38 denaturation cycles at 95°C for 30 s, primer annealing at 60°C for 30 s, and a primer extension phase at 72°C for 30 s. Upon the completion of the cycling steps, a final extension step at 72°C for 7 min was conducted before the reaction mixture was stored at 4°C. Real-time PCR was then employed to determine the fold increase of NQO1 mRNA in each of the primary breast tumors relative to the paired adjacent non-tumor tissue taken from the same patient. Double-stranded DNA specific expression was tested by the comparative Ct method using 2-ΔΔCt. Primers were as follows: NQO1 5′-GGC AGA AGA GCA CTG ATC GTA-3′, and 5′-TGA TGG GAT TGA AGT TCA TGG C-3′; GAPDH 5′-CAT CAC CAT CTT CCA GGA GCG-3″, and 5′-TGA CCT TGC CCA CAG CCT TG-3′. Expression data were normalized to the geometric mean of GAPDH to control the variability in expression levels. All experiments were performed in triplicate.

### Statistical analysis

Statistical analyses were performed using SPSS 17.0 software. Correlation between NQO1 expression and clinicopathological characteristics was evaluated using the χ2 test and Fisher’s exact tests. Disease-free survival (DFS) and 10-year overall survival (OS) after tumor removal were calculated using the Kaplan-Meier method, and differences in survival curves were analyzed using the Log-rank tests. Multivariate analysis was performed using the Cox proportional hazards regression model on all significant characteristics measured for univariate analysis. *P* < 0.05 was considered statistically significant.

## Results

### NQO1 mRNA and protein expression in breast cancers

**Figure 1:**
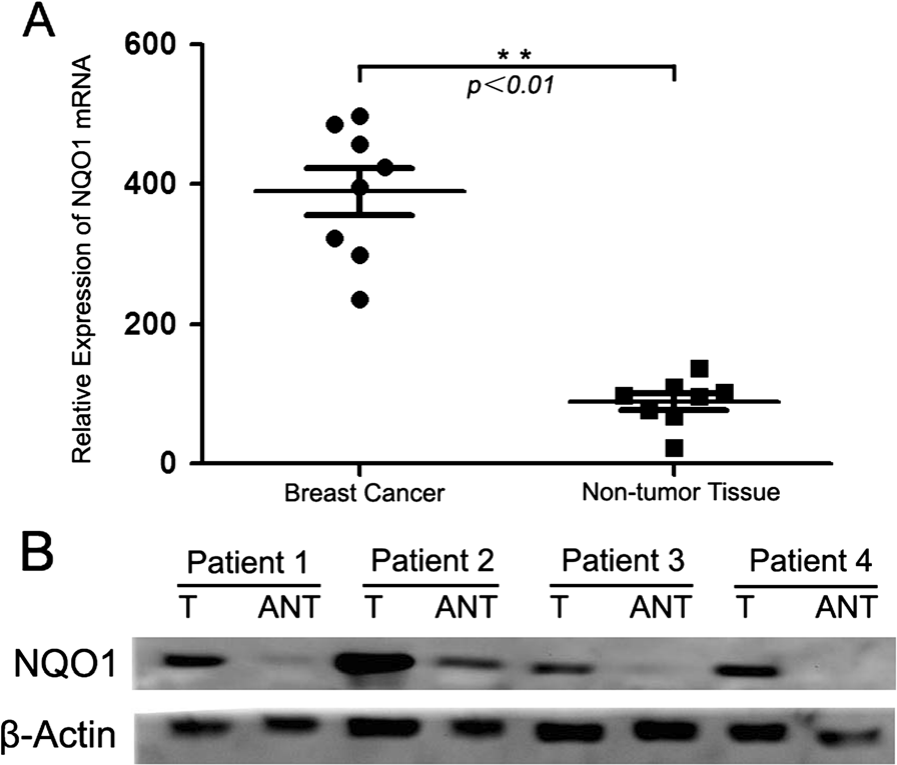
**Overexpression of NQO1 mRNA and protein in breast cancer tissues.** Expression of NQO1 mRNA and protein in breast cancers tissues (T) and adjacent non-tumor tissues (ANT) were examined by qPCR **(A)** and western blotting **(B)**. Data in **(A)** represent fold change of relative NQO1 mRNA expression normalized to GAPDH levels. Error bars represent the standard deviation of the mean (SD) calculated from three parallel experiments. *P < 0.05.

NQO1 mRNA levels were examined in eight pairs of breast cancers and adjacent non-tumor breast tissues using qRT-PCR. The results revealed that the relative mRNA expression level of NQO1 was significantly upregulated in cancers compared with adjacent non-tumor tissues (Figure 1A). Western blot data also demonstrated that NQO1 protein was highly expressed in breast cancer tissues compared with adjacent non-tumor tissues (Figure 1B).

**Figure 2:**
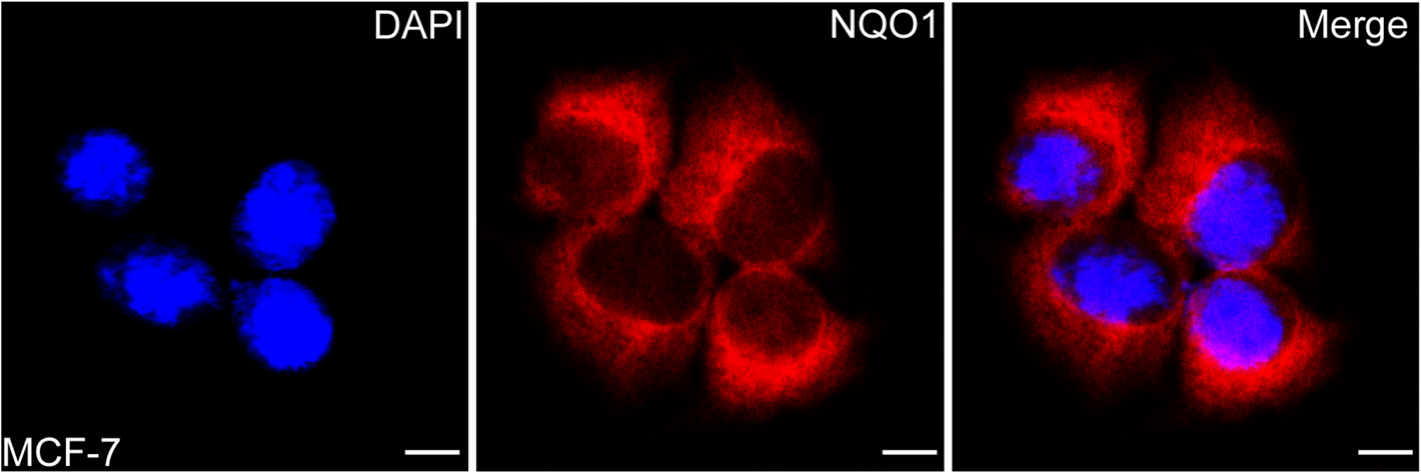
**Immunofluorescent staining of NQO1 in MCF**-**7 human breast cancer cells.** NQO1 protein located in the cytoplasm of breast cancer cells (red indicates NQO1 staining; Blue indicates DAPI).

To determine the subcellular localization of NQO1 protein, IF staining for NQO1 protein was performed in MCF-7 breast cancer cells. The staining results clearly showed that NQO1 protein is mainly located in the cytoplasm in MCF-7 breast cancer cells (Figure 2).

**Figure 3:**
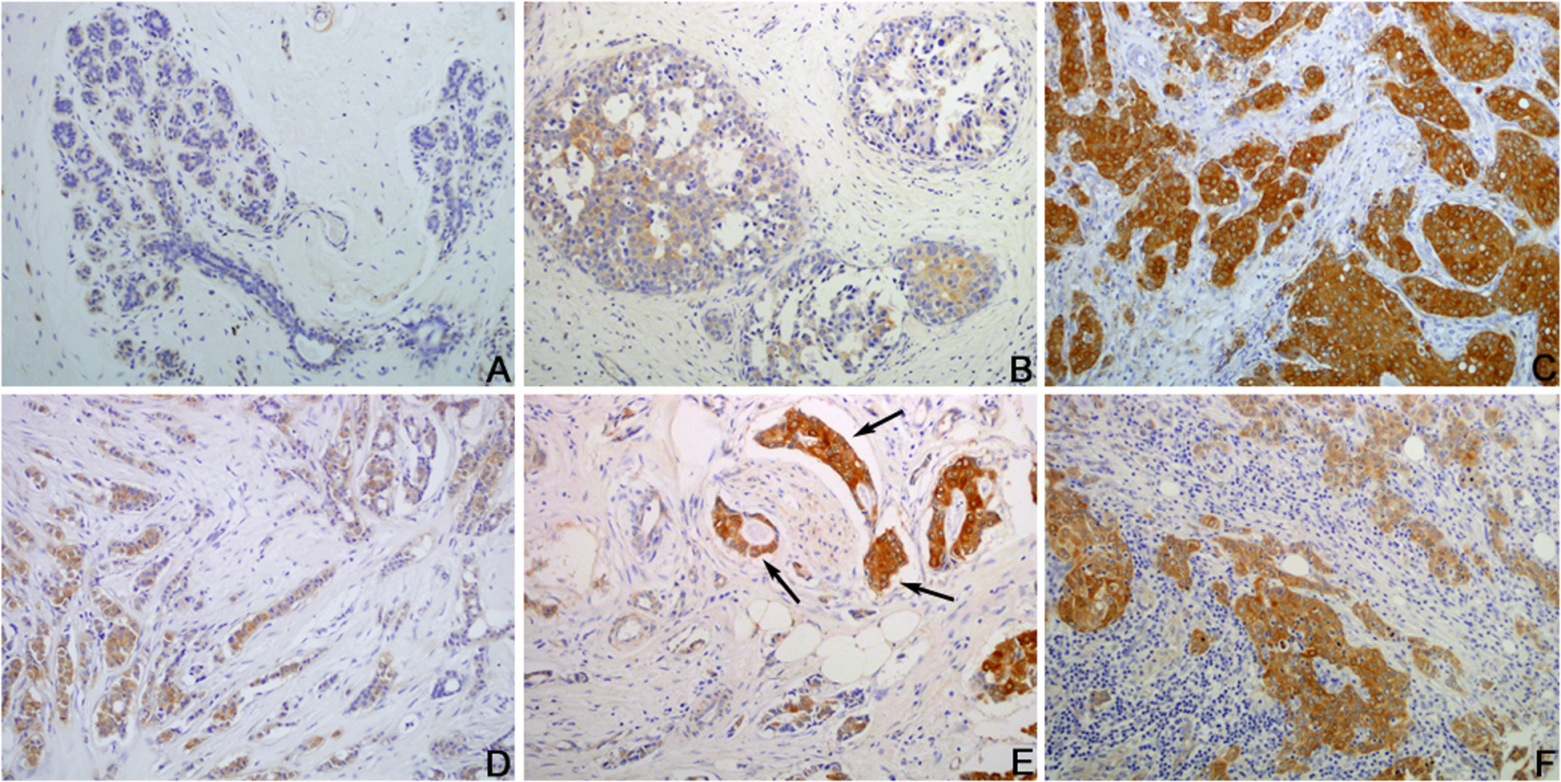
**Immunohistochemical staining for NQO1 protein expression. (A)** NQO1 staining is negative in non-tumor tissue. **(B)** Weakly positive NQO1 protein signals in breast hyperplasia. **(C)** Strongly positive NQO1 protein signal in breast cancer cases with metastasis. **(D)** Weakly positive NQO1 protein signal in invasive ductal breast cancers without metastasis. **(E)** Strongly positive NQO1 protein in the cancer cells metastatic to blood vessels (*arrows*). **(F)** Strongly positive NQO1 protein signal in the metastatic cancer loci in lymph node. Original magnification, A: ×100; B–F: ×200.

IHC staining also showed that NQO1 protein is mainly located in the cytoplasm of breast cancer cells (Figure 3). The positive rate of NQO1 protein expression was 84.7% (149/176) in breast cancers, which was significantly higher than that in hyperplasia (36.7%, 8/22) and adjacent non-tumor tissues (30.8%, 16/52) (*P* < 0.001). Similarly, the strongly positive rate of NQO1 expression was 61.9% (109/176) in breast cancers, which was also significantly higher than that in hyperplasia (13.6%, 3/22) and adjacent non-tumor tissues (13.5%, 7/52) (*P* < 0.001). More importantly, the positive rate of NQO1 protein in DCIS was also significantly higher (51.1%, 23/45) than hyperplasia (36.7%, 8/22) and adjacent non-tumor tissues (30.8%, 16/52) (Table 2). These data suggest that overexpression of NQO1 may be an early event that can be detected during the early stage of breast cancer.

**Table 2:** NQO1 expression in breast cancers

**Diagnosis**	**No. of cases**	**Positive cases**			**Positive cases rates**	**Strongly positive rates**
			**-**	**+**	**++**	**+++**		
Breast cancers	176	27	40	62	47	84.7%**	61.9%**
DCIS	45	22	9	10	4	51.1%*	31.1%*
Hyperplasia	22	14	5	3	0	36.7%	13.6%
Adjacent non-tumor	52	36	9	7	0	30.8%	13.5%

DCIS: ductal carcinoma *in situ*.

Positive rate: percentage of positive cases with +, ++, and +++ staining score. Strongly positive rate: (high-level expression) percentage of positive cases with ++ and +++ staining score.

*p<0.05 and **p<0.01 compared with non-tumor tissues.

### Clinicopathological significance of NQO1 protein overexpression in breast cancers

To evaluate the role of NQO1 protein in breast cancer progression, the correlation between NQO1 expression and clinical features of patients was analyzed. As summarized in Table 1, there were no significant correlations between the expression level of NQO1 protein and patient age, menopausal status, tumor size, ER levels or PR levels in patients with breast cancer. However, the strongly positive rate of NQO1 protein was significantly higher in Grade 2 and Grade 3 breast cancers than in Grade 1 cases (*P* = 0.004), and it was also higher in breast cancers with lymph node metastasis than in cases without metastasis (*P* = 0.005). In addition, overexpression of NQO1 showed a correlation with the clinical stage of breast cancer, which was higher in advanced stage (stage III–IV) breast cancers than in early stage (stage I–II) cases (*P* = 0.008). Furthermore, the strongly positive rate of NQO1 protein was higher in cancer cases with high Her2 expression compared to those with low Her2 expression.

### Association between NQO1 expression and prognosis of breast cancer patients

Univariate analysis demonstrated that histological grade (*P* = 0.004), clinical stage (*P* = 0.008), LN metastasis (*P* = 0.005), Her2 expression levels (*P* = 0.019), and NQO1 expression status were significantly associated with DFS and 10-year OS in patients with breast cancer (Table 3). These data suggest that NQO1 could be a valuable prognostic factor in breast cancer. Further multivariate analysis using the Cox proportional hazards model revealed that NQO1 overexpression emerged as a significant independent prognostic factor for survival along with clinical stage and Her2 expression in breast cancer (*P* = 0.040).

**Table 3:** Univariate and multivariate survival analyses (Cox regression model) of various factors in 176 breast cancer patients

**Factors**	**B**	**SE**	**Wald**	**HR (95%CI)**	***P *** **value**
**Univariate**					
Age	0.2650.265	0.153	2.991	1.303 (0.095–1.758)	0.084
Menopausal status	0.219	0.154	2.037	1.245 (0.921–1.683)	0.154
Tumor size	0.283	0.154	3.389	1.328 (0.982–1.795)	0.066
Histological grade	0.218	0.099	4.843	1.244 (1.024–1.510)	0.028
Clinical stage	1.017	0.169	36.097	2.766 (1.985–3.855)	0.000
LN metastasis	0.382	0.158	5.858	1.465 (1.075–1.996)	0.016
ER	0.190	0.153	1.525	1.209 (0.895–1.633)	0.217
PR	0.114	0.154	0.548	1.121 (0.829–1.515)	0.459
Her2	0.550	0.155	12.600	1.733 (1.279–2.437)	0.000
NQO1	0.447	0.157	8.055	1.563 (1.148–2.128)	0.005
**Multivariate**					
Histological grade	0.207	0.109	3.629	1.230 (0.994–1.521)	0.057
Clinical stage	0.906	0.175	26.929	2.475 (1.758–3.485)	0.000
LN metastasis	0.222	0.168	1.756	1.249 (0.889–1.736)	0.185
Her2	0.394	0.161	5.990	1.484 (1.082–2.035)	0.014
NQO1	0.372	0.181	4.216	1.450 (1.017–2.067)	0.040

B: Coefficient; SE: standard error; Wald: Waldstatistic; HR: hazard ratio.

**Figure 4:**
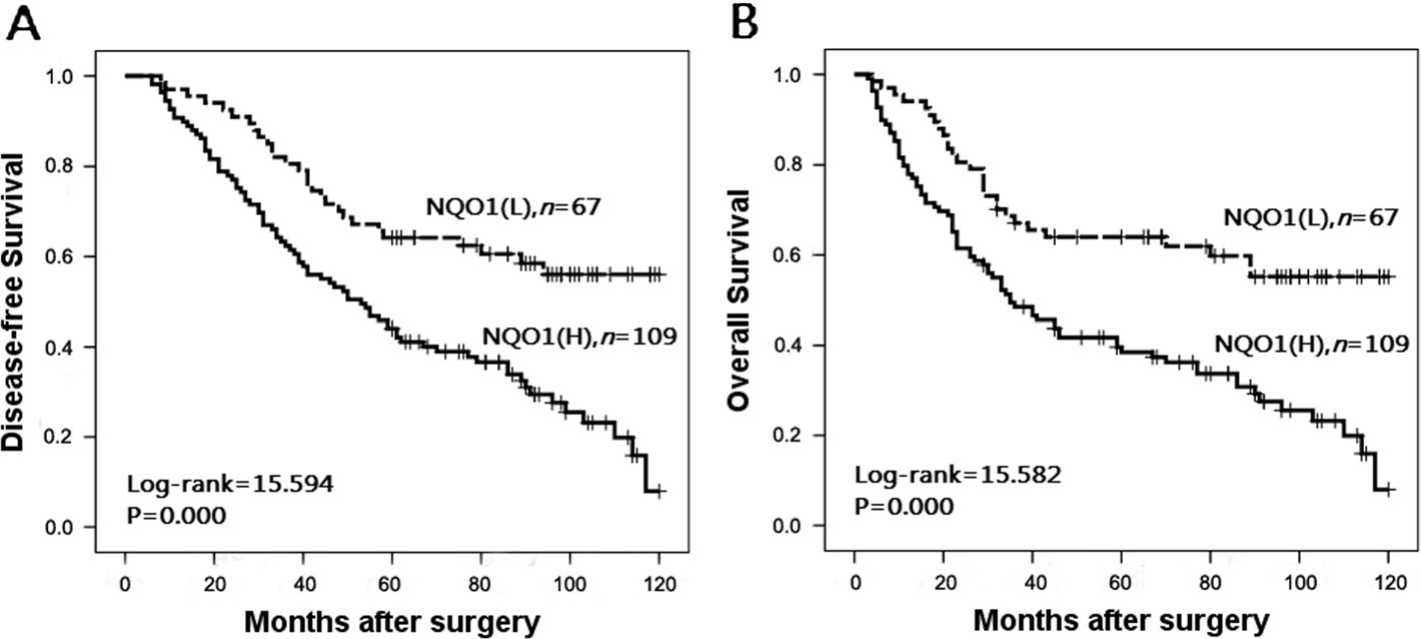
**Kaplan**–**Meier survival curves in patients with high and low NQO1 expression. (A)** and **(B)** show comparison of DFS and 10-year OS, respectively, in NQO1 low-expression (L) and high-expression (H) patients.

**Figure 5:**
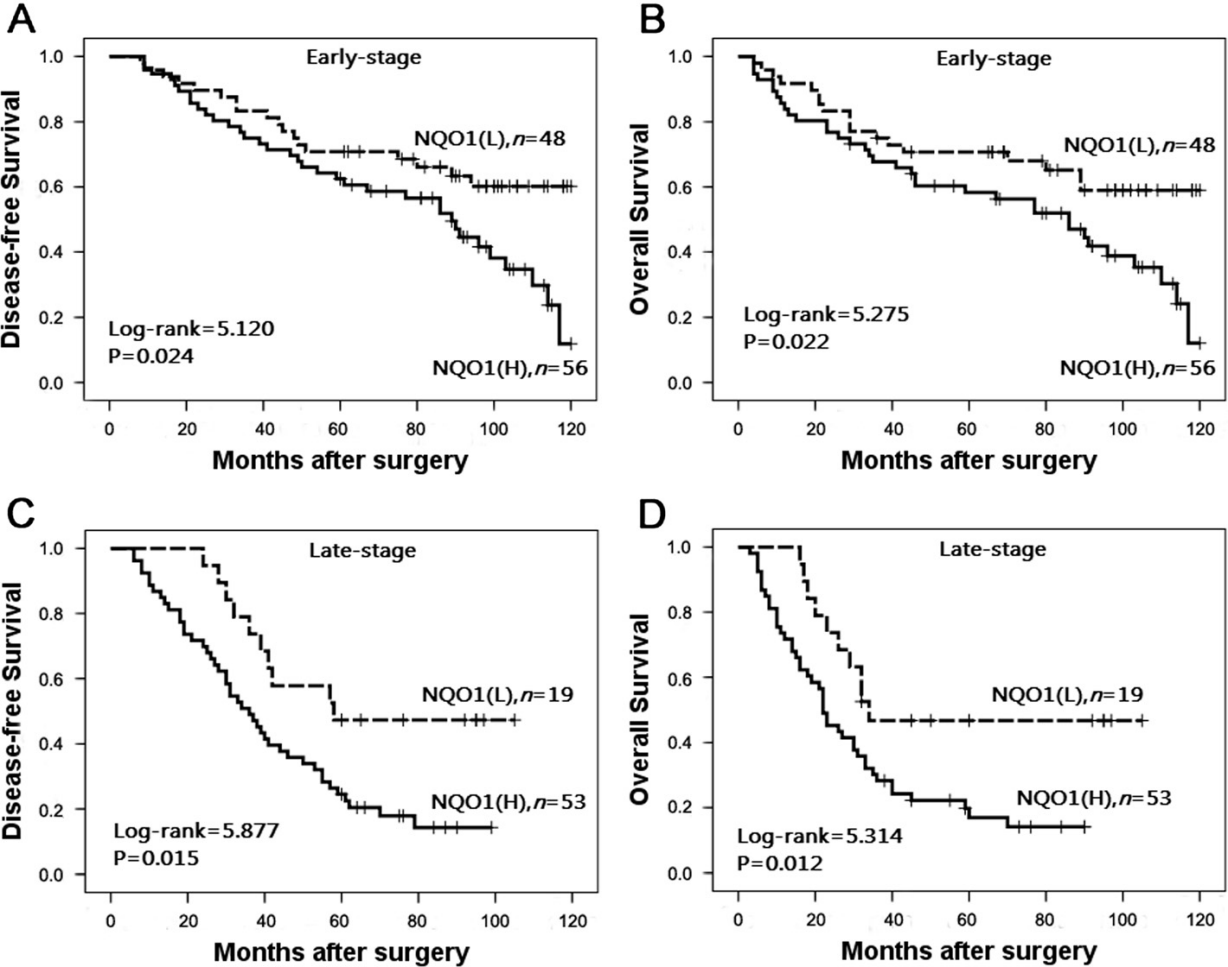
**Kaplan**–**Meier survival curves of in early and late stage patients. (A)** and **(B)** show comparison of DFS and 10-year OS, respectively, in NQO1 (L) and (H) patients of early stage. **(C)** and **(D)** show comparison of DFS and 10-year OS, respectively in NQO1 (L) and (H) patients of late stage.

**Figure 6:**
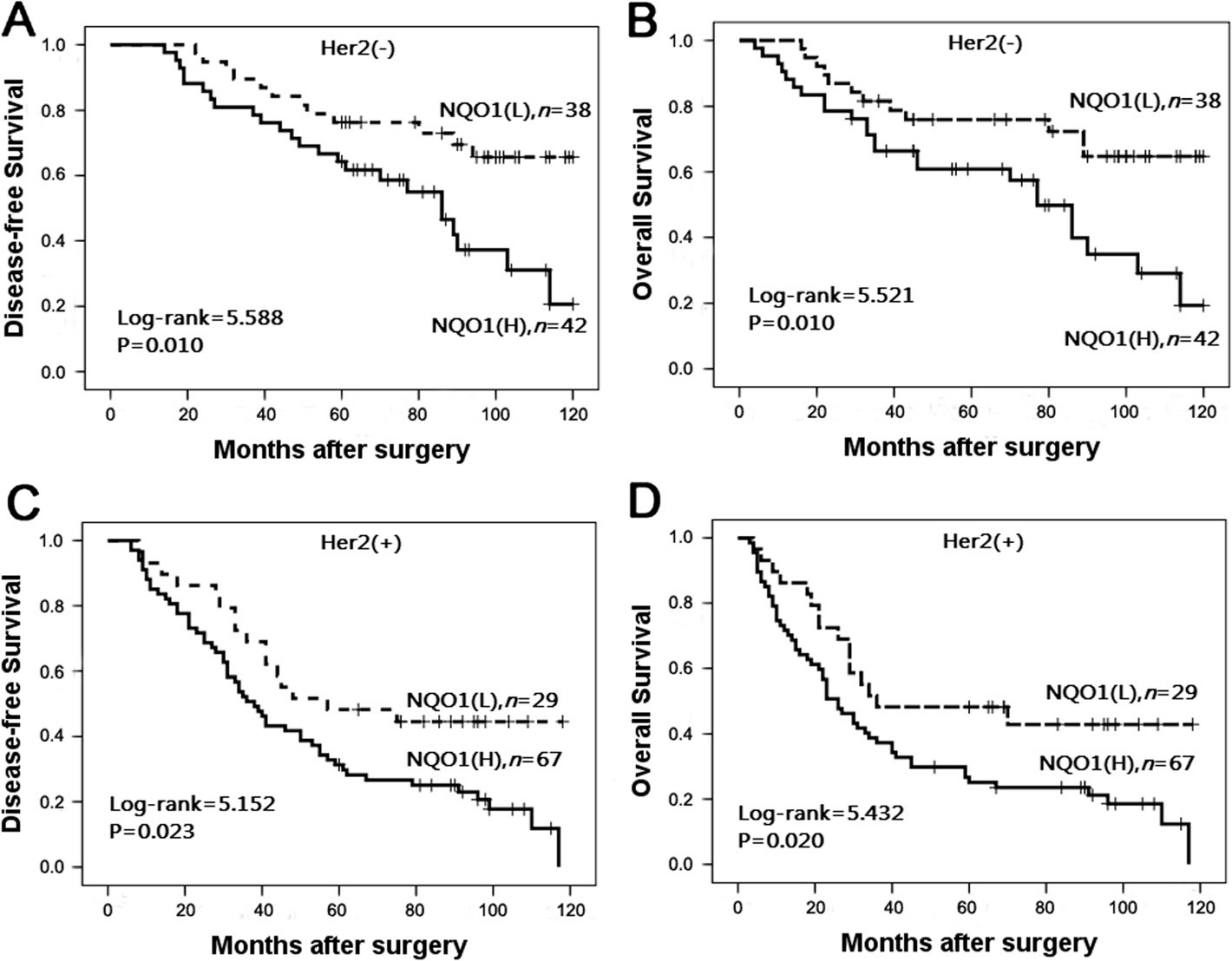
**Kaplan**–**Meier survival curves in patients with Her2 positive and negative expression. (A)** and **(B)** show comparison of DFS and 10-year OS, respectively, in NQO1 (L) and (H) patients with Her2 negative expression. **(C)** and **(D)** show comparison of DFS and 10-year OS, respectively, in NQO1 (L) and (H) patients with Her2 positive expression.

To further substantiate the importance of high NQO1 expression in breast cancer progression, we analyzed DFS and 10-year OS of 176 breast cancer cases using the Kaplan–Meier method and found that patients with high NQO1 expression had lower DFS and 10-year OS than those with low NQO1 expression (both *P* < 0.0001) (Figure 4). In addition, the expression of NQO1 was strongly associated with DFS and 10-year OS rates of patients with both early-stage tumors (P = 0.024) and late-stage tumors (*P* = 0.015) (Figure 5). Similarly, for patients with either Her2 low or high expression, high NQO1 expression showed significantly worse DFS and 10-year OS than those with low NQO1 expression (*P* = 0.010 and *P* = 0.023, respectively) (Figure 6).

## Discussion

NQO1 was first identified by Ernster and Navazio in the late 1950s [21]. After decades of research, considerable data has demonstrated that NQO1 can protect against natural and exogenous quinones. NQO1 expression in most human tissue types also suggests that it may function primarily in an antioxidant capacity in these cells. Notably, NQO1 is also upregulated in various mammalian cancers. Lyn-Cook et al. demonstrated that NQO1 expression is higher in pancreatic adenocarcinomas compared to non-tumor tissues [22].

Wakai et al. demonstrated strong IHC staining of NQO1 in intrahepatic cholangiocarcinoma (ICC), whereas the non-tumor bile ducts and liver parenchyma were weakly stained. Cheng et al. showed that NQO1 expression is significantly increased in primary melanomas compared with dysplastic nevi and this may occur in the initiation stage of melanoma development [23]. A recent focus of current research has been the identification of polymorphisms in NQO1, which have been demonstrated as an increased risk of some tumors. Ouerhani et al. reported that the NQO1C609T genotype was overrepresented in acute lymphoblastic leukemia patients and was associated with an aggravating effect compared to the reference group with NQO1 C609C genotype [24]. Jamieson et al. reported the NQO1 SNP (rs1800566) was associated with a poorer outcome and a lower likelihood of having a treatment delay [25]. These findings indicated that NQO1 may have roles in carcinogenesis and tumor progression. However, the clinicopathological significance of NQO1 protein expression in breast cancer is less clear.

In this study, we performed IHC staining of NQO1 protein in breast cancer tissue. In agreement with previous studies [13,15], we found that staining of NQO1 is mainly localized in the cytoplasm, and these observations were consistent with our IF staining results in MCF-7 breast cancer cells. Compared with adjacent non-tumor tissues, NQO1 protein was found to be significantly up-regulated in breast cancer using IHC. Western blot and qRT-PCR results also demonstrated that the mRNA and protein levels of NQO1 in four cases of fresh breast cancer samples were elevated compared with the adjacent non-tumor tissues. Furthermore, our IHC results showed that the positive rate of NQO1 protein in DCIS was also significantly higher than either hyperplasia or adjacent normal tissues, indicating that NQO1 upregulation may occur in the initiation stage of breast cancer progression. These findings suggest that NQO1 protein level might be used as an early diagnostic indicator of this disease.

Despite the strong association between NQO1 expression and cancer, there have been few reports of NQO1 protein expression-based outcomes in tumor patients. Mikami K et al. reported that the expression and enzyme activity of NQO1 is not only upregulated in colon cancer cell lines and colorectal tumors, but also significantly greater in tumors with nodal metastases than those without metastases [26], while Gan et al. reported higher expression of NQO1 protein in lower-grade and superficial bladder tumors compared with high-grade and invasive tumors [27]. In the present study, we found that the NQO1 expression level was markedly associated with histological grade (P = 0.004), clinical stage (*P* = 0.008), LN metastasis (*P* = 0.005) and Her2 expression levels (*P* = 0.019), suggesting that NQO1 upregulation promotes the invasion and/or metastasis of breast cancer cells. These finding indicate that NQO1 might be useful as a poor prognostic biomarker of breast cancer. Moreover, Buranrat et al. demonstrated a significant association between high level of NQO1 expression and short overall survival time of cholangiocarcinoma patients, which raises the exciting possibility of using NQO1 as a tumor marker [14]. Additionally, an association was found by Awadallah et al. between high level of NQO1 expression and short overall survival of pancreatic cancer patients [15]. In our previous study, we found that high level of NQO1 protein significantly associated with shortened survival of patients with gastric adenocarcinoma [28]. However, the alternative hypothesis seems to be true with low NQO1 expression evaluated by IHC in intrahepatic cholangiocarcinoma (ICC) cases predicting poor prognosis [29]. The conflicting conclusions may be due to the different study populations, which also highlights the need to evaluate the biomarkers under relevant circumstances. In the present study, univariate survival analysis revealed that tumor histological grade, clinical stage, LN metastasis, Her2 expression level and NQO1 expression status are all significantly related with DFS and10-year OS rates of patients with breast cancer (*P* < 0.05). Further multivariate survival analysis showed that NQO1 expression was one of the independent prognostic factors, along with tumor clinical stage and Her2 status. Moreover, finding tumor-selective therapies for breast cancer is of utmost importance. Our study with NQO1 protein expression in breast cancers also indicated that as an exploitable cancer target, NQO1 might improve patient management and outcome by personalized therapy. A comprehensive analysis of the molecular mechanism of NQO1 involved in the tumorigenesis and progression of breast cancer is essential.

## Conclusions

In summary, NQO1 plays a key role in the progression of breast cancer, and high level of NQO1 protein is strongly associated with advanced stage, lymph node metastasis, Her2 overexpression and shortened survival of patients with breast cancer. The high proportion and prognostic value of NQO1 expression suggests that NQO1 may be a significant biomarker and a potential therapeutic target for patients with breast cancer.

## Competing interests

The authors declare that they have no competing interests.

## Authors’ contributions

YY and YZ contributed equally to this work. All authors read and approved the final manuscript.
